# Secondary autoimmune hypothalamitis with severe memory impairment 7 years after the onset of diabetes insipidus due to lymphocytic hypophysitis: a case report

**DOI:** 10.1186/s12883-022-02891-z

**Published:** 2022-09-29

**Authors:** Takahiro Asada, Shintaro Takenoshita, Mayuko Senda, Koichiro Yamamoto, Ryo Sasaki, Fumio Otsuka, Seishi Terada, Norihito Yamada

**Affiliations:** 1 Department of Neuropsychiatry, Okayama University Hospital, 2-5-1 Shikata-cho, Kita-ku, Okayama, 700-8558 Japan; 2 Department of General Medicine, Faculty of Medicine, Dentistry and Pharmaceutical Sciences, Okayama University, Okayama, Japan; 3 Department of Neurology, Faculty of Medicine, Dentistry and Pharmaceutical Sciences, Okayama University, Okayama, Japan; 4 Department of Neuropsychiatry, Faculty of Medicine, Dentistry and Pharmaceutical Sciences, Okayama University, Okayama, Japan

**Keywords:** Lymphocytic hypophysitis, Autoimmune hypophysitis, Autoimmune hypothalamitis, Cognitive dysfunction, Memory impairment

## Abstract

**Background:**

Autoimmune hypothalamitis is a very rare neuroendocrine disorder that causes central diabetes insipidus, headache, visual impairment, and sometimes cognitive impairment. Autoimmune hypothalamitis may occur in association with autoimmune hypophysitis, including lymphocytic hypophysitis, or in isolation. It is not known whether autoimmune hypothalamitis and autoimmune hypophysitis are consecutive diseases.

**Case presentation:**

A 52-year-old woman developed autoimmune hypothalamitis 7 years after developing central diabetes insipidus due to lymphocytic hypophysitis, resulting in severe memory impairment. High-dose intravenous methylprednisolone therapy improved her cognitive function and decreased the size of the lesion.

**Conclusion:**

This case presented a unique clinical course, with a long period of time between the onset of autoimmune hypopituitaritis and the development of autoimmune hypothalamitis.

## Background

Lymphocytic hypophysitis is one type of autoimmune hypophysitis (AH), a rare neuroendocrine disorder that causes chronic inflammation of the pituitary gland [1, 2]. AH can cause central diabetes insipidus (CDI) and hypopituitarism. Autoimmune hypothalamitis (AHT) is an inflammation of the hypothalamus anatomically continuous with the pituitary gland, and shows pathological features similar to autoimmune hypophysitis [3–5]. AHT may result in diabetes insipidus (DI), headache, visual impairment, hypopituitarism, and sometimes cognitive dysfunction. AH and AHT occur as overlapping or isolated cases, and it is not well known whether they are contiguous diseases; their long-term course remains unclear [4, 6–10]. We report a case of AHT that developed 7 years after developing CDI due to lymphocytic hypophysitis, and resulting in severe memory impairment that was recovered with high-dose intravenous methylprednisolone treatment.

## Case presentation

The patient was a 52-year-old female whose chief complaint was memory impairment. She had had two caesarean deliveries, at the ages of 22 and 26 years, with massive hemorrhage during the first delivery and insufficient lactation after the second delivery. She had no family history of neuroendocrine disorders. She was a nursing home employee. At the age of 44 years, she visited the first hospital with a sudden onset of thirst and a chief complaint of polydipsia of more than 6 l per day and polyuria of more than 3 l per day. Her body mass index (BMI) was 23.5. The main laboratory values were plasma osmolality 286 mOsm/kg H2O, urine specific gravity 1.002, serum sodium concentration 141 mEq/dl, and decreased plasma vasopressin level <1.2 pg/ml. She was diagnosed with CDI due to polyuria (urinary volume > 4–5 ml/kg/h), low urine specific gravity, and extremely low vasopressin level (< 1.2 pg/ml) relative to plasma osmolality. Magnetic resonance imaging (MRI) showed hypertrophy with contrast-enhanced effects in the whole pituitary gland and the pituitary stalk after gadolinium administration. Other laboratory values (normal ranges) were adrenocorticotropic hormone (ACTH) 10.1 pg/ml (7.2–63.3), thyroid-stimulating hormone (TSH) 13.85 μIU/ml (0.35–5.0), free thyroxine (FT4) 0.8 ng/dl (0.97–1.69), prolactin (PRL) 29.4 ng/ml (4.91–29.32), growth hormone (GH) 0.25 ng/ml (0.13–9.88), insulin-like growth factor (IGF-1) 112 ng/ml (88–229), luteinizing hormone (LH) 0.10 mIU/ml (1.0-95.6), follicle-stimulating hormone (FSH) 0.89 mIU/ml (1.7–21.5), cortisol 9.5 μg/dl (4.0–18.3), and hemoglobin A1c (HbA1c) 5.5%, and negative for serum autoantibodies such as antinuclear antibodies and pituitary cell antibodies. A pituitary load test showed ACTH, PRL, and TSH hyperresponsiveness and revealed hypothalamic hypofunction. A transsphenoidal biopsy of the pituitary gland showed lymphocytic infiltration (data not shown). On the other hand, immunostaining for immunoglobulin G4 was negative, and there was no evidence of sarcoidosis or neoplastic lesions. She was diagnosed histologically as having lymphocytic hypophysitis. Administration of 10 mg per day hydrocortisone reduced the pituitary swelling, and l-deamino-8-D-arginine vasopressin (DDAVP) by a nasal spray reduced polydipsia and polyuria, and improved abnormalities in the pituitary load test. She continued to receive levothyroxine supplementation, 5 mg per day hydrocortisone, and DDAVP orally.

At the age of 46 years, she developed a highly fatty liver. She underwent arginine and GH-releasing peptide 2 (GHRP2) tests and was diagnosed with GH deficiency; recombinant human GH (rhGH) was started. At the age of 48, rhGH was discontinued because she developed diabetes mellitus. At this point, she had no cognitive impairment and was working without hindrance as a caregiver.

**Fig. 1:**
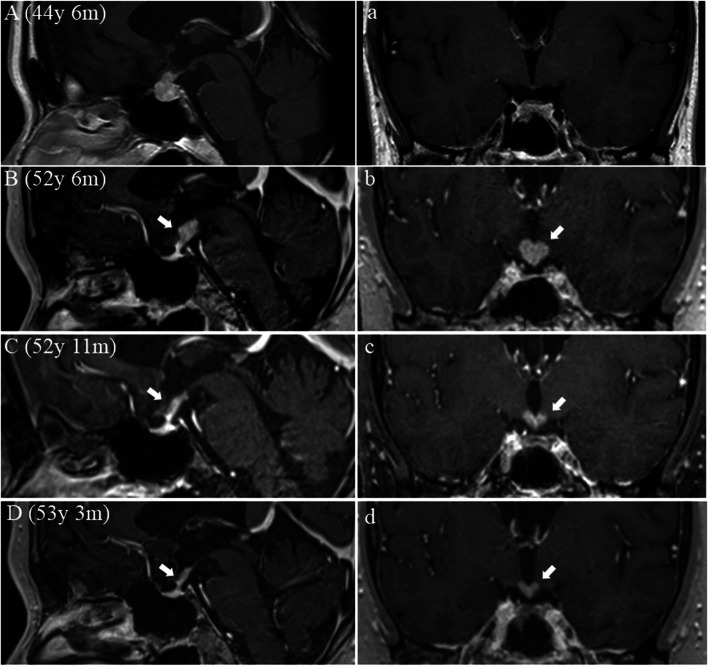
Head magnetic resonance imaging (sagittal, coronal, T1-weighted image with gadolinium). A, a When she developed central diabetes insipidus, hypertrophy with contrast-enhanced effects in both adenopituitary and neuropituitary and thickening of the pituitary stalk were seen. B, b When she developed memory impairment, contrast-enhanced effects in the pituitary stalk and the mammillary bodies of the hypothalamus were seen. After the first course of high-dose methylprednisolone pulse treatment (HDMPT) (C, c) and after the second course of HDMPT (D, d), the volume of the lesion was significantly reduced

At the age of 51 years and 11 months, she developed symptoms of memory impairment such as saying the same thing over and over and completely forgetting events that had occurred minutes before. Within 3 months, she was no longer able to work as a caregiver. Her memory impairment further progressed, and she became unable to manage her finances. At the age of 52 years and 4 months, she was referred to our hospital on suspicion of young-onset dementia and admitted. On admission, her BMI was 41.7. She was clearly conscious and able to comprehend in the moment but had significant memory impairment. She had thirst, osteoporosis, hyperuricaemia, dyslipidaemia, and amenorrhea. Her mood was not depressed, and she had no cranial nervous system abnormalities such as visual impairment. MRI showed the loss of the posterior pituitary bright spot (PPBS) on T1-weighted images, and contrast-enhanced effects in the pituitary stalk and the mammillary bodies of the hypothalamus (Fig. 1). Laboratory values (normal) were ACTH 25.2 pg/ml (7.2–63.3), TSH 0.29 μIU/ml (0.35–5.0), FT4 0.95 ng/dl (0.97–1.69), PRL 16.7 ng/ml (4.91–29.32), GH 0.07 ng/ml (0.13–9.88), IGF-1 41.80 ng/ml (88–229), LH <0.3 mIU/ml (1.0–95.6)，FSH 0.7 mIU/ml (1.7–21.5), cortisol 10.3 μg/dl (4.0–18.3), and HbA1c 6.3%, and negative for serum autoantibodies such as antinuclear antibodies. Electroencephalography was normal, and a spinal fluid examinations (protein, cell count, IgG index, tau protein, phosphorylated tau protein, amyloid beta 42, amyloid beta 40, anti-NMDA receptor antibody, and anti-VGKC receptor antibody) were normal. The Wechsler Memory Scale-Revised (WMS-R) showed a marked decline in memory (Table 1) [11]. Based on the MRI abnormalities, it was assumed that lymphocytic hypophysitis had spread to the hypothalamus, and three courses of high-dose methylprednisolone pulse treatment (HDMPT), methylprednisolone 500 mg/day, for 3 days, were administered. Three months after HDMPT, her MRI showed a reduced lesion (Fig. 1) but no apparent change in WMS-R scores (Table 1). At the age of 52 years and 7 months, she was discharged and continued prednisolone orally. After the HDMPT, her diabetes mellitus did not worsen, but the degree of her memory impairment did not change.

**Table 1:** Course of memory impairment

	52 y 4 m		52 y 7 m		53 y 3 m
WMS-R					
General Memory	77	HDMPT 500 mg/day 3 day, 3 course	70	HDMPT 1000 mg/day 3 day, 2 course	82
Verbal Memory	78		72		77
Visual Memory	85		79		100
Attention/Concentration	87		88		96
Delayed Recall	< 50		< 50		78

*Abbreviations*: *HDMPT* High-dose methylprednisolone pulse treatment, *WMS-R* Wechsler Memory Scale-Revised

At the age of 53 years and 1 month, she was hospitalized again, and treated with two courses of an increased dose of HDMPT, methylprednisolone 1000 mg/day, for 3 days. After HDMPT treatment, lesions of the MRI were further reduced and her WMS-R scores showed clear improvement (Fig. 1, Table 1). She was discharged and continued prednisolone orally. In this case, a biopsy of the hypothalamus was not performed because of its invasiveness, but the clinical course in which the lesions of the MRI shrank in response to HDMPT with concomitant improvement in cognitive function led to the diagnosis of AHT.

## Discussion and conclusion

We report a case of AHT with memory impairment 7 years after the onset of lymphocytic hypophysitis with CDI as the main symptom, in which treatment with HDMPT resulted in imaging and functional improvement.

CDI is an endocrine disorder resulting in decreased antidiuretic hormone (vasopressin) and polyuria. The prevalence of CDI is estimated at 1 in 25,000 [12]. Approximately 30 to 50% of CDI cases are idiopathic [13, 14]. Further, it has been suggested that most idiopathic CDI develops via autoimmune processes in the hypothalamus and pituitary gland [3, 14, 15].

Lymphocytic hypophysitis, one of the causes of CDI, is a form of AH characterized by pituitary enlargement and pituitary destruction due to lymphocytic infiltration [2]. Lymphocytic hypophysitis is very rare, with an estimated annual incidence of 1 in 9 million people, but it may also be underdiagnosed [1]. MRI shows pituitary enlargement resembling a pituitary adenoma, diffuse and homogeneous contrast enhancement of the anterior pituitary gland, and loss of the PPBS [2, 16, 17]. The cause of lymphocytic hypophysitis is unknown, but it is more common in women, often develops during the last trimester of pregnancy or postpartum. Lymphocytic hypophysitis causes headaches, hypopituitarism, adrenal insufficiency, hypothyroidism, hyperprolactinemia, and excess GH. In the long term, the pituitary gland atrophies. Glucocorticoids and azathioprine have been used for treatment in many cases [2, 18, 19]. Inflammation has been reported to spread to the dura mater and cavernous sinus, but there have been very few reports of inflammation progressing to the hypothalamus over a long period of time [3, 10, 20].

AHT causes focal or diffuse infiltration of lymphocytes into the hypothalamus, histologically resembling lymphocytic hypophysitis [2, 5, 7, 9, 10, 21]. AHT causes headache, visual impairment, CDI, and hypopituitarism. The number of reported cases is very small, and the prevalence is unknown. MRI of AHT produces iso-intense T1-weighted images, hyper-intense in T2-weighted images, and loss of the PPBS in the hypothalamus [6, 10]. Due to the similarity of pathological findings, clinical symptoms, and course of treatment, some have argued that AHT is a subtype of AH [7, 10]. However, it is not clear whether AHT and AH are consecutive diseases or not because isolated AHT without pituitary inflammation has also been reported [4–9]. There have been no reports of cognitive dysfunction when inflammation is limited to the pituitary gland, but there have been reports of cognitive dysfunction (memory or attention) in some cases of AHT [3, 5, 10, 22]. It is speculated that the cognitive decline in hypothalamic inflammation is related to the fact that the mammillary body of the hypothalamus is a part of the Papez circuit, which is a neural network related to memory [23, 24]. The concept of hypothalamic syndrome (HS) has been proposed for a combination of memory impairment, obesity, and diabetes mellitus resulting from diseases of the hypothalamus [25]. In this case, long-term use of hydrocortisone may have contributed to the development of obesity and diabetes mellitus, in addition to HS. When memory impairment occurs in patients with severe obesity and diabetes mellitus, the possibility of obstructive sleep apnea syndrome (OSAS) should also be considered, but in this case, there were no observable signs of OSAS.

Although case reports of AHT are rare and the pattern of its clinical course has not yet been clarified, two cases like the present study, in which a woman developed AHT and memory impairment about 10 years after developing CDI, have been reported. Dow et al. reported a case that developed lymphocytic hypophysitis with CDI as the main symptom at the age of 26 years, followed 10 years later by AHT with cognitive dysfunction [3]. They reported that the patient gained partial improvement in cognitive function after treatment with HDMPT, methylprednisolone 250 mg/day for 3 days. In addition, Bertulli et al. reported a case that developed CDI at the age of 55 years, followed 12 years later by AHT and cognitive dysfunction [5]. That case was diagnosed as AHT by biopsy. They treated the patient with HDMPT, methylprednisolone 1000 mg/day for 3 days, and azathioprine 50 mg/day, but there was no improvement.

Although there are only a few reports of cognitive dysfunction due to AHT, HDMPT may affect recovery of cognitive function. There have been several reports of AHT occurring about 10 years after the onset of CDI, which may be one pattern of the clinical course of AHT.

## Data Availability

The datasets used and/or analyzed during the current study are available from the corresponding author on reasonable request.

## Abbreviations

AH: Autoimmune hypophysitis
AHT: Autoimmune hypothalamitis
CDI: Central diabetes insipidus
DDAVP: l-deamino-8- D-arginine vasopressin
HDMPT: High-dose methylprednisolone pulse treatment
HS: Hypothalamic syndrome
OSAS: Obstructive sleep apnea syndrome
PPBS: Posterior pituitary bright spot on T1-weighted images
WMS-R: Wechsler Memory Scale-Revised
